# Supplementary material for: Switches, stability and reversals in the evolutionary history of sexual systems in fish
Source: Nat Commun. 2022 May 30;13:3029. doi: 10.1038/s41467-022-30419-z (PMC9151764; doi:10.1038/s41467-022-30419-z)
Supplement: Supplementary file 1 — Supplementary Information [file 41467_2022_30419_MOESM1_ESM.pdf]

Supplementary information for

Switches, stability and reversals in the evolutionary history of sexual systems in fish

Supplementary tables

Supplementary Table 1. Results of the RJ-MCMC Multistate analysis in *BayesTraits* of sexual systems as a binary trait: gonochorism (G) or hermaphroditism (H). For each posterior distribution, we report the effective sample size (ESS), the mean and 95% high posterior density (95-HPD) intervals, the mode, and the percentage of models in which the parameter is estimated as zero. Analysis based on 4614 extant teleost species (G: n = 4320; H: n = 294)

**Transition rates**	**ESS**	**Mean**	**95-HPD**	**Mode**	**% Zero**
G → H	1600	0.034	0.021 – 0.047	0.032	**0**
H → G	1600	0.826	0.608 – 1.038	0.823	**0**
**Root probabilities**					
G	1600	66.0	59.1 – 74.1	65.1	**0**
H	1600	34.0	25.9 – 40.9	34.9	**0**

Supplementary Table 2. Results of phylogenetic generalized least square (PGLS) model of longevity (year; log_{10} transformed), controlled for allometry (maximum length, in cm; log_{10} transformed; age at first maturity (year; log_{10} transformed) per each sex (♂: male; ♀: female), controlling for allometry (length at first maturity in cm; log_{10} transformed); and male gonadosomatic index (GSI; log_{10} transformed), controlling for allometry (male length at first maturity in cm; log_{10} transformed) across sexual systems: gonochorism (G); protogyny (PG); protandry (PA). For each independent variable we report the parameter estimate (Beta), t-statistics (T), *P*-value (*P*; two-sided test), and the model statistics including the degrees of freedom (df), the maximum likelihood estimation of the phylogenetic signal (λ) and R^2. Significant differences are indicated in bold. See Supplementary Table 3 for sexual system and sex-specific data

Variable		Beta	T	*P*	Df	Model statistics	
**Dependent**	**Independent**					**λ**	$\mathbf{R^2}$
Longevity	Max length	0.680	19.968	**$< 2^{-16}$**	3; 640	0.864	0.388
	Sexual system - PA[1]	-0.043	-0.417	0.677			
	Sexual system - PG[1]	0.129	2.193	**0.029**			
	Sexual system - PG[2]	0.172	1.538	0.124			
Age at first maturity ♂	Length at maturity ♂	0.602	8.360	**4.086^{-14}**	3; 149	0.785	0.325
	Sexual system - PA[1]	-0.090	-0.956	0.340			
	Sexual system - PG[1]	0.068	0.774	0.440			
	Sexual system - PG[2]	0.159	1.434	0.154			
Age at first maturity ♀	Length at maturity ♀	0.536	8.062	**1.408^{-13}**	3; 166	0.829	0.282
	Sexual system - PA[1]	0.016	0.117	0.907			
	Sexual system - PG[1]	-0.014	-0.167	0.867			
	Sexual system - PG[2]	-0.029	-0.217	0.828			
GSI ♂	Length at maturity ♂	0.041	0.236	0.814	3; 51	0.000	0.376
	Sexual system - PA[1]	0.164	1.197	0.237			
	Sexual system - PG[1]	-0.513	-4.631	**2.547^{-05}**			
	Sexual system - PG[2]	-0.678	-4.582	**2.999^{-05}**			

Supplementary Table 3. Sample sizes for longevity (year), maximum length (cm), age at first maturity (years), length at first maturity (cm) and gonadosomatic index (GSI) for each sexual system with sex-specific male (♂) and female (♀) data, when available. In italics data not used in the analyses. G = gonochorism; PG = protogyny; PA = protandry; SH = simultaneous hermaphroditism; BD = bidirectional hermaphroditism

**Life-history traits**	**Sex**	**G**	**PG**	**PA**	**Total for analyses**	***SH***	***BD***
Longevity		758	69	17	844	*7*	*3*
Maximum length		2612	167	20	2799	*28*	*11*
Age at first maturity	♂	259	15	9	283	*1*	-
	♀	282	30	5	317	*2*	-
Length at first maturity	♂	305	42	15	362	*9*	-
	♀	297	36	10	343	*2*	-
GSI	♂	44	38	15	97	*3*	-

Supplementary Table 4. Number of species used to study the evolutionary transitions among different sexual systems. Note that androdioecious species are not included in the analyses

**Sexual system**	**2 state** [G, H]	**4 state** [G, PG, PA, SH]
Gonochorism [G]	4320	4320
Hermaphroditism [H]	294*	
Protogyny [PG]		196
Protandry [PA]		36
Simultaneous hermaphroditism [SH]		46
Total	4614	

*Includes 16 species of bidirectional sequential hermaphrodites, which were not included in other analyses due to their small sample size when added as a separate category.

Supplementary figure

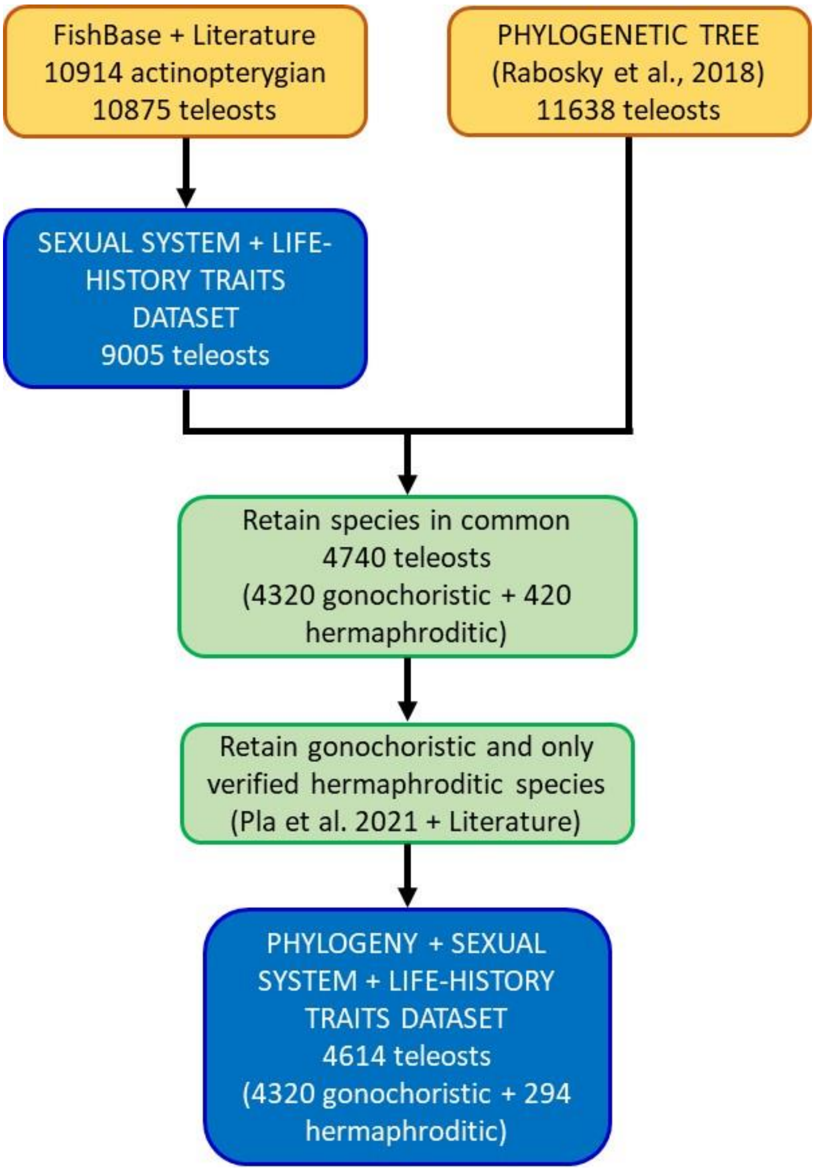

Supplementary Figure 1. Diagram of the data acquisition and curation followed in this study.
