# Supplementary material for: Switches, stability and reversals in the evolutionary history of sexual systems in fish
Source: Nat Commun. 2022 May 30;13:3029. doi: 10.1038/s41467-022-30419-z (PMC9151764; doi:10.1038/s41467-022-30419-z)
Supplement: Supplementary file 3 — Description of Additional Supplementary Files [file 41467_2022_30419_MOESM3_ESM.pdf]

Description of Additional Supplementary Files

File Name: Supplementary Data 1
Description: Excel file listing all the species used with information on sexual system and life history plus reference list to the primary literature supporting functional hermaphroditism in fish.
