# Supplementary material for: Switches, stability and reversals in the evolutionary history of sexual systems in fish
Source: Nat Commun. 2022 May 30;13:3029. doi: 10.1038/s41467-022-30419-z (PMC9151764; doi:10.1038/s41467-022-30419-z)
Supplement: Supplementary file 5 — Reporting Summary [file 41467_2022_30419_MOESM5_ESM.pdf]

nature portfolio

Corresponding author(s): Francesc Piferrer, Isabella Capellini

Last updated by author(s): Apr 27, 2022

Reporting Summary

Nature Portfolio wishes to improve the reproducibility of the work that we publish. This form provides structure for consistency and transparency in reporting. For further information on Nature Portfolio policies, see our Editorial Policies and the Editorial Policy Checklist.

Statistics

For all statistical analyses, confirm that the following items are present in the figure legend, table legend, main text, or Methods section.

n/a	Confirmed	
☐	☒	The exact sample size (*n*) for each experimental group/condition, given as a discrete number and unit of measurement
☒	☐	A statement on whether measurements were taken from distinct samples or whether the same sample was measured repeatedly
☐	☒	The statistical test(s) used AND whether they are one- or two-sided<br>*Only common tests should be described solely by name; describe more complex techniques in the Methods section.*
☐	☒	A description of all covariates tested
☐	☒	A description of any assumptions or corrections, such as tests of normality and adjustment for multiple comparisons
☐	☒	A full description of the statistical parameters including central tendency (e.g. means) or other basic estimates (e.g. regression coefficient) AND variation (e.g. standard deviation) or associated estimates of uncertainty (e.g. confidence intervals)
☐	☒	For null hypothesis testing, the test statistic (e.g. *F*, *t*, *r*) with confidence intervals, effect sizes, degrees of freedom and *P* value noted<br>*Give P values as exact values whenever suitable.*
☐	☒	For Bayesian analysis, information on the choice of priors and Markov chain Monte Carlo settings
☒	☐	For hierarchical and complex designs, identification of the appropriate level for tests and full reporting of outcomes
☒	☐	Estimates of effect sizes (e.g. Cohen's *d*, Pearson's *r*), indicating how they were calculated

Our web collection on statistics for biologists contains articles on many of the points above.

Software and code

Policy information about availability of computer code

Data collection	R package rfishbase (https://www.rdocumentation.org/packages/rfishbase/versions/4.0.0)
Data analysis	Open source software used: for Bayesian analysis BayesTraits v3 available at http://www.evolution.rdg.ac.uk/BayesTraitsV3.0.2/BayesTraitsV3.0.2.html; for PGLS analysis R package caper.

For manuscripts utilizing custom algorithms or software that are central to the research but not yet described in published literature, software must be made available to editors and reviewers. We strongly encourage code deposition in a community repository (e.g. GitHub). See the Nature Portfolio guidelines for submitting code & software for further information.

Data

Policy information about availability of data

All manuscripts must include a data availability statement. This statement should provide the following information, where applicable:

- Accession codes, unique identifiers, or web links for publicly available datasets
- A description of any restrictions on data availability
- For clinical datasets or third party data, please ensure that the statement adheres to our policy

Data were extracted from FishBase (https://fishbase.org), from Fish tree of life (https://fishtreeoflife.org) and from the primary literature. The dataset used for the analyses and associated references are submitted along with the manuscript as Supplementary Data 1. All data collected and analysed in this study are included in this published article and its Supplementary information and Data source files.

Field-specific reporting

Please select the one below that is the best fit for your research. If you are not sure, read the appropriate sections before making your selection.

☐ Life sciences ☐ Behavioural & social sciences ☒ Ecological, evolutionary & environmental sciences

For a reference copy of the document with all sections, see nature.com/documents/nr-reporting-summary-flat.pdf

Ecological, evolutionary & environmental sciences study design

All studies must disclose on these points even when the disclosure is negative.

Study description	Phylogenetic comparative study of the evolutionary history of sexual system and association with life history traits in teleost fish.
Research sample	4614 extant teleost species, with information on heir sexual system out of an initial dataset with a total of 10914 actinopterygian species representative of all actinopterygians of the world. Of the 4614 teleosts, 4320 were gonochoristic and 294 hermaphroditic (protogynous: n = 196; protandrous: n = 36; bidirectional sex change: n = 16; simultaneous hermaphroditism: n = 46). A subsample of these have life history data as reported in Supplementary Table 3.
Sampling strategy	All available data extracted from FishBase for sexual system and life history strategies; species used were those also present in the phylogeny. Sample sizes and procedure in Methods.
Data collection	Data from FishBase for all sexual system; checked and species matched to phylogeny. Details and sample sizes in Supplementary Information.
Timing and spatial scale	Global Scale; timing not relevant
Data exclusions	Data for species not in the phylogeny was discarded
Reproducibility	MCMC analyses in triplicates
Randomization	N/A
Blinding	N/A

Did the study involve field work? ☐ Yes ☒ No

Reporting for specific materials, systems and methods

We require information from authors about some types of materials, experimental systems and methods used in many studies. Here, indicate whether each material, system or method listed is relevant to your study. If you are not sure if a list item applies to your research, read the appropriate section before selecting a response.

Materials & experimental systems

n/a	Involved in the study	
☒	☐	Antibodies
☒	☐	Eukaryotic cell lines
☒	☐	Palaeontology and archaeology
☒	☐	Animals and other organisms
☒	☐	Human research participants
☒	☐	Clinical data
☒	☐	Dual use research of concern

Methods

n/a	Involved in the study	
☒	☐	ChIP-seq
☒	☐	Flow cytometry
☒	☐	MRI-based neuroimaging
